# Dietary polyvinyl alcohol and alginate nanofibers ameliorate hyperglycemia by reducing insulin and glucose-metabolizing enzyme levels in rats with streptozotocin-induced diabetes

**DOI:** 10.14202/vetworld.2021.847-853

**Published:** 2021-04-09

**Authors:** Muhammad Suryadiningrat, Devia Yoanita Kurniawati, Agung Mujiburrahman, Muhammad Thohawi Elziyad Purnama

**Affiliations:** Department of Veterinary Science, Division of Veterinary Anatomy, Faculty of Veterinary Medicine, Universitas Airlangga, Surabaya 60115, Indonesia

**Keywords:** alginate nanofibers, blood glucose, diabetes, insulin, serum

## Abstract

**Background and Aim:**

Dietary management and antidiabetic drugs are used as therapies for diabetic patients worldwide. Alginate nanofibers were developed as a digestible food product that provides sufficient calories. This study aimed to evaluate the effect of polyvinyl alcohol (PVA) and alginate nanofibers on weight gain, blood glucose levels, and insulin and other serum parameters in diabetic rats.

**Materials and Methods:**

A total of 24 male Wistar rats were divided into six groups: (C−) Control group, (C+) diabetic rats, (T1) diabetic rats + fasting treatment for 12 h, (T2) diabetic rats + nanofibers *ad libitum*, (T3) diabetic rats + metformin + nanofibers *ad libitum*, and (T4) diabetic rats + metformin. All groups were treated for 21 days. Weight gain was evaluated by comparing initial and final weights. Blood glucose levels were evaluated weekly. Serum parameters were also evaluated at the end of the study. All variables were analyzed statistically using analysis of variance followed by Tukey’s *post hoc* test (p<0.05).

**Results:**

The T2, T3, and T4 groups showed a significant increase in weight compared to that of the C+ and T1 groups. The T3 group had the lowest blood glucose level of all groups at the end of the study. In the serum evaluation, the T2 and T3 groups showed a significant decrease compared to the C+ group for the following variables: Alanine aminotransferase (ALT), alkaline phosphatase (ALP), gamma-glutamyltransferase (GGT), creatinine, tumor necrosis factor-alpha (TNF-α), and interleukin-1β (IL-1β). In contrast, the T2 group showed a significant decrease compared to the T3 group for aspartate aminotransferase and insulin levels.

**Conclusion:**

PVA and alginate nanofibers can modulate obesity, reduce blood glucose levels, and reduce serum levels of insulin, ALT, ALP, GGT, creatinine, TNF-α, and IL-1β in diabetic rats.

## Introduction

Estimates of the number of patients with Type 1 diabetes continue to increase worldwide [1]. This phenomenon is associated with dietary fat, polysaccharides, and fiber [2]. Dietary fiber can increase satiety by controlling energy intake and modifying digestive function; different forms of fiber have various effects on the rate and extent of starch and lipid digestion [3]. The amount of food consumed is a major determinant of postprandial hyperglycemia. The glycemic load depends on the food glycemic index (GI) and the amount consumed [4]. In other studies, Type 2 diabetes due to insulin resistance was associated with obesity, aging, lifestyle, and protein anabolic responses [5].

The GI was designed as a guide for food selection for people with diabetes. Lower GI foods produce a relatively low glycemic response after consumption compared with that produced by higher GI foods [4]. The role of alginates in food intake and glycemic regulation has been explored [6]. Alginate is derived from the cell walls of brown seaweed, often *Sargassum* spp. This compound is a heteropolysaccharide formed from the monomers mannuronic acid and guluronic acid. The alginate content in *Sargassum* spp. is 30-40% of its dry matter [7]. Nanotechnology in food concerns a special class of colloidal particles of 1-1000 nm in size, and one of these forms is a fiber called a nanofiber. A nanofiber is defined as a fiber with a diameter under 1 mm [8]. Alginate-based nanofiber synthesis can improve gel stability, fiber composition, and printed materials [9].

In the present work, we characterized polyvinyl alcohol (PVA) and alginate nanofibers using proximate analysis, scanning electron microscopy (SEM), and Fourier-transform infrared (FTIR) spectroscopy. Furthermore, we analyzed the effect on weight gain, blood glucose levels, and serum parameters including insulin, in rats with streptozotocin-induced diabetes. Evaluation of the acquired data revealed a potential alternative diet in a diabetic rat model.

## Materials and Methods

### Ethical approval

The *in vivo* study was conducted on male Wistar rats with the goal of minimizing stress, animal suffering, and the number of collected samples. Ethics certificate No. 2.KE.086.05.2019 was issued by the Faculty of Veterinary Medicine, Universitas Airlangga.

### Study period and location

This study was conducted for 3 months (March-May 2019). Male Wistar rats were reared in the Laboratory Animal Facility, Program Studi di Luar Kampus Utama, Banyuwangi, Universitas Airlangga. The alginate nanofiber preparation was carried out at the Physics Laboratory, Faculty of Science and Technology, Universitas Airlangga. Proximate analysis was performed at the Nutrition Laboratory, Faculty of Veterinary Medicine, Universitas Airlangga. SEM and FTIR analyses were performed at the Engineering Laboratory, Institut Teknologi Sepuluh Nopember, Surabaya. Laboratory examinations were performed at Gamma Scientific Biolab, Malang, East Java.

### Synthesis and characterization of alginate nanofibers

*Sargassum* spp. as a raw material was collected from the seaweed market in Banyuwangi, East Java. A total of 10 kg of *Sargassum* spp. were dried in a Memmert UN110 oven at 120°C for 36 h. Next, the dry matter was crushed into a powder. The synthesis of alginate nanofibers was carried out by mixing solutions of 15% PVA and 1.5% alginate at 80°C while stirring at 800 rpm for 30 min [10]. Then, the two polymers were mixed at a ratio of 8:2. The mixture of PVA and alginate was loaded into a syringe pump and electrospinning was started to produce nano-sized fibers. In electrospinning, an electric voltage is applied to the solution to induce a free charge on a nano-sized stream of polymer solution that is sprayed on the collector [8]. The electric voltage used in this study was 18 kV and the collector distance was 12 cm from the tip of the syringe pump [10]. The morphological characterization of the synthesized nanofibers was carried out using SEM and FTIR. This evaluation aimed to determine the physical form, distribution of the polymers, size of the material, and functional groups of mixed compounds [11]. Meanwhile, to analyze the nutrient content in 100 g of alginate nanofibers, a proximate analysis was performed [12].

### Experimental animals

A total of 24 male Wistar rats were divided into six groups: C− was the control group, C+ was rats with streptozotocin-induced diabetes, T1 was diabetic rats with fasting treatment for 12 h, T2 was diabetic rats with a diet of alginate nanofibers *ad* *libitum*, T3 was diabetic rats with metformin (400 mg/kg body weight [BW]/day peroral treatment [13]) and a diet of alginate nanofibers *ad libitum*, and T4 was diabetic rats with metformin (400 mg/kg BW/day). All groups were treated for 21 days. During the experimental period, rats were fed a balanced commercial diet (Charoen Pokphand, Indonesia) and pure water *ad libitum*, except for groups T2 and T3 which did not receive the commercial diet.

The rats were acclimatized for 1 week before treatment. Diabetes was induced by intraperitoneal injection of streptozotocin (Sigma, USA) at a dose of 30 mg/kg BW. Three days after streptozotocin administration, 0.5 mL blood samples were collected from the tail vein. The diabetic rats were confirmed to have blood glucose levels above 250 mg/dL [14]. On days 0, 7, 14, and 21, 0.5 mL blood samples were collected from the tail vein for blood glucose evaluation using the GOD/POD Kit (Span Diagnostics Ltd., India) at 505 nm. Initial and final weights were also measured to evaluate weight gain. After the study period, all rats were euthanized, and blood samples were collected to obtain serum for biochemical analysis [15].

### Biochemical analysis

Blood from a cardiac puncture (1.5 mL) was collected in a plain serum microtube and allowed to clot for 10-15 min in an icebox, followed by centrifugation in a Benchmark Scientific MC-24 at a speed of 1500 rpm for 10 min. The supernatant was used to measure the following variables: Aspartate aminotransferase (AST), alanine aminotransferase (ALT), alkaline phosphatase (ALP), gamma-glutamyltransferase (GGT), creatinine, insulin, tumor necrosis factor-alpha (TNF-α), and interleukin-1β (IL-1β) [16]. All variables were measured using an ELISA kit (Korain Biotech Co. Ltd., Shanghai, China) according to the manufacturer’s instructions.

### Statistical analysis

All data are expressed as mean ± standard deviation. Weight gain, AST, ALT, ALP, GGT, creatinine, insulin, TNF-α, and IL-1β were analyzed with one-way analysis of variance (ANOVA) followed by Tukey’s *post hoc* test. Blood glucose level data were analyzed with univariate ANOVA followed by Tukey’s *post hoc* test. Values were considered significantly different at p<0.05. Statistical analysis was performed using SPSS v.25 software (IBM, Armonk, NY, USA).

## Results

### Characterization of alginate nanofibers with proximate analysis, SEM, and FTIR

The proximate analysis of alginate nanofiber showed the following composition: 95.27% dry matter, 1.31% ash, 4.62% protein, 3.36% fat, 28.14% crude fiber, 1.09% calcium, and 2.60 kcal/g total calories (Table-1). Protein and fat levels are affected by photocatalytic processes during electrospinning [17]. However, the composition of the fiber produced by electrospinning can be considered a suitable dietary formula. Fiber above 22% and total calories above 1.8 kcal/g are standard for balanced animal feed [18].

**Table-1:** Evaluation of alginate nanofibers using proximate analysis.

Ingredients	% in 100 g
Dry matter	95.27
Ash	1.31
Protein	4.62
Fat	3.36
Crude fiber	28.14
Calcium	1.09
Total calories	2.60 kcal/g

**Figure-1:**
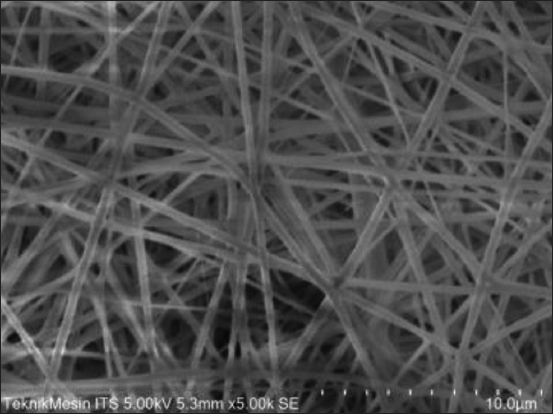
Characterization of alginate nanofibers using scanning electron microscopy.

Characterization using SEM was carried out to determine the size and distribution of the produced 8:2 PVA and alginate nanofibers. SEM was performed at a voltage of 5.00 kV and 5000×. The average size of the fiber formed was 321 nm; the fiber was evenly distributed with no beads found (Figure-1). The prevention of bead formation during the electrospinning process demonstrated successful nanofiber synthesis [19]. Limiting the number of beads can improve the diameter, morphology, polymer distribution, and density during electrospinning [20].

**Figure-2:**
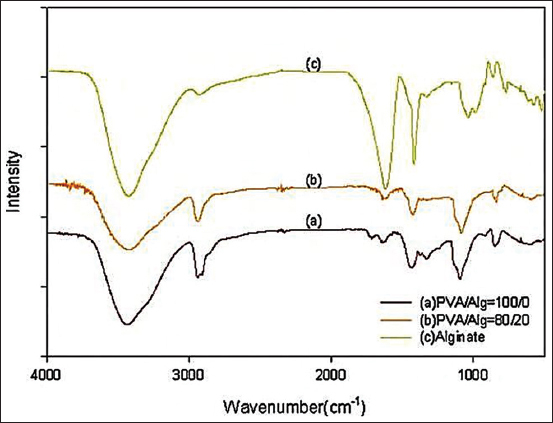
Characterization of polyvinyl alcohol and alginate using Fourier transform infrared. The formulation (b) used in this study.

Functional group tests using FTIR showed that the PVA and alginate nanofibers were stable at an 8:2 ratio compared to fibers with only alginate or PVA (Figure-2). Such polymer mixtures are reported to have biocompatible properties and stable functional groups [21].

### Effect of alginate nanofibers on weight gain

**Figure-3:**
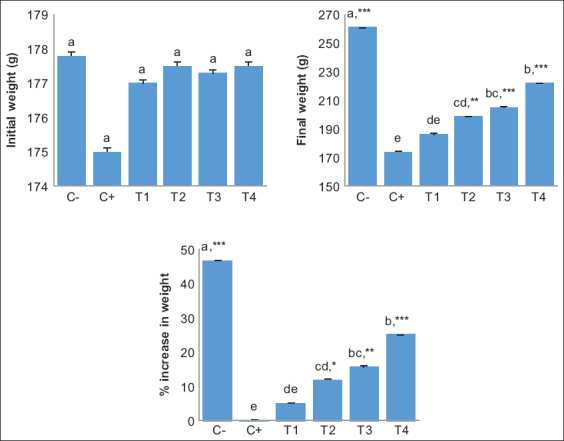
Values are expressed in mean±standard deviation (n=4 animals for each six groups). One-way analysis of variance was carried out followed by *post hoc* Tukey multiple comparisons test. Values are represented statistically when ^a,b,c,d,e^ in comparison with the C- group; *p<0.05, **p<0.01, and ***p<0.001, in comparison with the C+ group.

In general, the weight increased in the T2, T3, and T4 groups. The T2 (11.9±4.36^cd,^*), T3 (15.7±2.75^bc,^**), and T4 groups (25.1±7.50^b,^***) showed a significant increase in weight compared to that of the C+ (0.1±1.19^e^) and T1 groups (5.2±4.66^de^). There were no significant differences in weight gain between the T2 (11.9±4.36^cd,^*) and T3 groups (15.7±2.75^bc,^**). In addition, the change in the T3 group (15.7±2.75^bc,^**) was not significant compared with that in the T4 group (25.1±7.50^b,^***) (Figure-3).

### Effect of alginate nanofibers on blood glucose level

**Figure-4:**
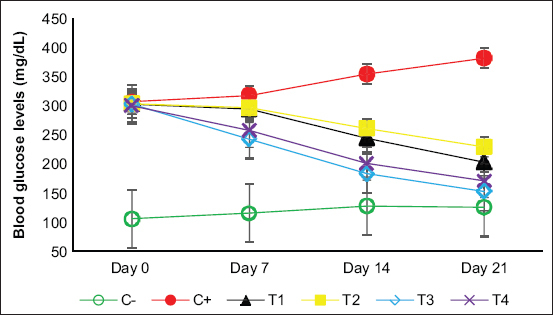
Line diagrams revealed the progress of blood glucose levels (mg/dL) on the day of 0, 7, 14, and 21, respectively. Values are expressed in mean±standard deviation (n=4 animals for each six groups).

For blood glucose, C+ showed a significant increase (381.5±11.15^e^) throughout the study compared to that of the other groups. On day 7, the glucose level in the T3 group (242.5±29.41^b,^***) showed a significant decrease compared to that in the C+ group (317.3±12.34^e^), and this decreased continuously (152.8±9.22^b,^***) until day 21. Meanwhile, the T3 group (183.3±12.45^b,^***) showed no significant difference compared to the other groups on day 14. The T3 group had the lowest blood glucose level (152.8±9.22^b,^***) of all treatment groups at the end of the study. Based on this evaluation, a significant improvement in hyperglycemia was revealed in the T3 group (Figure-4).

### Effect of alginate nanofibers on serum AST, ALT, ALP, GGT, creatinine, insulin, TNF-α, and IL-1β

**Figure-5:**
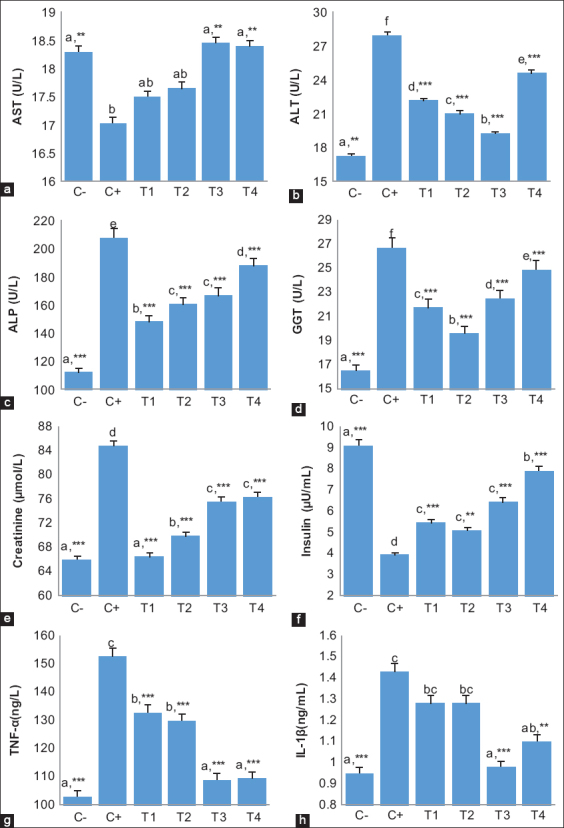
The level of serum (a) AST, (b) ALT, (c) ALP, (d) GGT, (e) creatinine, (f) insulin, (g) TNF-α, and (h) IL-1β in all groups, respectively. Values are expressed in mean ± SD (n=4 animals for each six groups). One-way analysis of variance was carried out followed by *post hoc* Tukey multiple comparisons test. Values are represented statistically when ^a,b,c,d,e,f,^ in comparison with C- group; *p<0.05, **p<0.01, and ***p<0.001, in comparison with the C+ group.

There was a significant increase in the AST level in the T3 (18.4±0.42^a,^**) and T4 groups (18.3±0.22^a,^**) compared to that in the C+ group (17.0±0.79^b^) (Figure-5a). In contrast, the ALT (19.5±0.51^b,^***), ALP (160.3±0.63^c,^***), and GGT (22.8±0.26^d,^***) levels showed a significant decrease in the T3 group compared to in the C+ group (27.9±0.13^f^, 207.5±7.57^e^, and 26.8±0.26^f^, respectively). In addition, the T4 group showed a significant increase in ALT (24.6±0.29^e,^***), ALP (187.6±0.49^d,^***), and GGT (24.9±0.22^e,^***) levels compared to those in the T3 group (Figure-5b-d).

There were significant decreases in the creatinine level in all treatment groups compared to that in the C+ group (84.8±2.54^a^). In particular, the level in the T1 group (66.5±0.70^e,^***) was significantly improved compared to that in the T2 (69.9±0.26^d,^***), T3 (75.1±0.39^c,^***), and T4 groups (76.4±0.51^b,^***) (Figure-5e).

The insulin level significantly increased in the T3 (8.3±0.51^b,^***) and T4 groups (7.9±0.17^b,^***) compared to that in all treatment groups. Moreover, the T3 group showed a slightly higher level than that in the T4 group (Figure-5f).

For cytokines, TNF-α and IL-1β were observed. There were significant decreases in the levels of TNF-α and IL-1β in the T3 (108.5±1.29^a,^*** and 1.0±0.08^a,^***) and T4 groups (109.5±1.29^a,^*** and 1.1±0.08^ab,^**) compared to those in the C+ group (152.8±5.56^c^ and 1.4±0.09^c^, respectively). In addition to this, the T3 (108.5±1.29^a,^***) and T4 groups (109.5±1.29^a,^***) showed a significant difference compared to the T1 (132.8±2.75^b,^***) and T2 groups (130.0±9.35^b,^***) in the level of TNF-α. However, the T4 group (1.1±0.08^ab,^**) showed no significant difference compared to the T1 (1.3±0.05^bc^) and T2 groups (1.3±0.05^bc^) in the level of IL-1β (Figure-5g and h).

## Discussion

Streptozotocin was used in this study to induce hyperglycemia and critically damage pancreatic β-cells. This drug can induce acute Type 2 diabetes and is characterized as an insulinotropic agent, demonstrating stable and long-lasting hyperglycemia [22]. Meanwhile, metformin is used to treat Type 2 diabetes by blocking the gluconeogenesis pathway, decreasing plasma triglycerides and low-density lipoprotein cholesterol levels, reducing systolic and diastolic blood pressure, and inducing vasoprotective effects [23]. Metformin reduces blood glucose levels by several mechanisms. The inhibitory pathways include directly stimulating glycolysis in peripheral tissue by increasing glucose release from the blood, reducing liver gluconeogenesis, slowing the absorption of blood glucose, reducing plasma glucose levels, and increasing insulin binding to receptors. The mechanism for reducing blood glucose levels does not depend on the presence of pancreatic β-cells [24].

Fiber intake modulates gastrointestinal physiology and normalizes bowel movement [25]. Dietary fiber is the portion of consumed food that is composed of carbohydrates that are resistant to digestion and absorption in the small intestine and fermented partially or completely in the large intestine. Dietary fiber is not hydrolyzed or digested by human digestive enzymes and includes hemicellulose, cellulose, lignin, oligosaccharides, pectin, and gum. Dietary fiber is classified as either soluble or insoluble dietary fiber. Soluble dietary fiber includes pectin and gum, which are found in fruits and vegetables. Insoluble dietary fiber includes cellulose, hemicellulose, and lignin, which are found in cereals and legumes [26]. Compared to high-calorie diets, low-calorie diets with high crude fiber content and low sugar and fat levels can reduce the risk of obesity [27].

It has been suggested that sufficient dietary fiber prevents excessive food intake and fat accumulation by decreasing the caloric density of the diet, slowing the rate of food ingestion, increasing the effort involved in eating, and promoting intestinal satiety [28]. Its role in maintaining blood glucose and cholesterol levels has been studied extensively worldwide [29]. Dietary fiber is able to absorb water and bind glucose, thereby reducing the availability of glucose in the blood. Dietary fiber also produces soluble complex carbohydrates and fiber to reduce carbohydrate digestibility. These conditions can alleviate increases in blood glucose [4].

Alginate oligosaccharides from seaweed can control the effects of hypoglycemia [30]. In one study conducted on rabbits, blood glucose levels after consumption of feed containing alginate were found to be significantly lower than the levels without alginate intake [31]. This fact is thought to be related to the ability of PVA and alginate to produce short-chain fatty acids (SCFAs), acetic acid, and propionic acid, which can help stimulate the insulin response in the liver [32]. The previous studies demonstrated the use of alginate as dietary fiber in the form of partially hydrolyzed polysaccharides [33]. A study on the clinical use of alginate in nutritional formulas was also the first to reveal its effect on the intestinal environment [34]. Alginate allegedly increases the absorption of monosaccharides, further affecting the total SCFA production. Alginates are likely to exert beneficial prebiotic effects on intestinal function through SCFA production [35]. Bioactive peptides in nanofiber alginate can reduce blood sugar levels if consumed long term. Stress in the endoplasmic reticulum plays a major role in obesity, insulin resistance, and Type 2 diabetes, which provides new evidence that nanofiber alginate induces a hypoglycemic effect through decreasing resistance to the liver insulin response and also reducing stress on the endoplasmic reticulum [36].

In addition, the treatment of overweight and diabetic rats by administration of pure fiber significantly reduced hyperglycemia, restored insulin sensitivity, resolved fatty liver disease, and increased insulin action in liver tissue [37]. The liver plays a major role in controlling glucose and insulin homeostasis in the pancreas compared to in fat and muscle tissue. The insulin signal in the liver is important for maintaining normal liver function [38]. Inhibition of hepatic glucose catabolism is a characteristic of metabolic syndrome, also known as insulin resistance syndrome, characterized by the elevation of serum liver AST, ALT, ALP, and GGT [39]. In the present study, a significant decrease was observed in ALT, ALP, and GGT levels after nanofiber alginate treatment. In contrast, an increase was found in the AST level. Fluctuation in serum AST and ALT associated with metformin metabolism in hepatocyte parenchyma cells before excretion in the urine [24].

Alginate nanofiber does not directly affect cytokine activity [40]. Normal level of insulin from pancreatic β-cells maintain the production of pro-inflammatory cytokines such as TNF-α and IL-1β [41]. The overall quantity of cytokines associated with apoptosis can be increased by cancer and aging. Moreover, TNF-α mediates various biological responses, including inflammation, infection, cell injury, and hyperglycemia [42]. In another study, nanofiber alginate acted as an immunostimulant by inhibiting the activity of TNF-α or IL-1β. The effect of TNF-α is initiated by cytokine receptor binding, which activates the main transcription factors, including nuclear factor kappa B. The activation then induces genes involved in the inflammatory response. Cells activated by cytokines may produce the same cytokines as paracrine signals or stabilize the signals through autocrine regulation [43].

## Conclusion

PVA and alginate nanofibers modulated obesity, alleviated blood glucose levels, and ameliorated reduced insulin in rats with streptozotocin-induced diabetes. A decrease was also observed in the levels of ALT, ALP, GGT, creatinine, and the cytokines TNF-α and IL-1β. Based on these findings, we recommend further study of dietary PVA and alginate nanofiber at varying doses. The glycosylated hemoglobin level also needs to be evaluated in future studies.

## Authors’ Contributions

MTEP supervised the study. MS and AM conducted the study. DYK performed *in vivo* analysis. MTEP did the statistical analysis of the data. MS and DYK prepared tables and figures. AM and DYK drafted the manuscript. MTEP revised the manuscript. All authors read and approved the final manuscript.
